# Polysomnographically mediated cognitive improvements in individuals with insomnia symptoms following continuous theta-burst stimulation of the default mode network

**DOI:** 10.3389/frsle.2024.1424083

**Published:** 2024-10-02

**Authors:** Alisa Huskey, Julia M. Fisher, Lindsey Hildebrand, David Negelspach, Kymberly Henderson-Arredondo, Samantha Jankowski, Salma I. Patel, Ying-Hui Chou, Natalie S. Dailey, William D. S. Killgore

**Affiliations:** ^1^Department of Psychiatry, College of Medicine, University of Arizona, Tucson, AZ, United States; ^2^BIO5 Institute, Statistics Consulting Laboratory, University of Arizona, Tucson, AZ, United States; ^3^Department of Medicine, University of Arizona Health Sciences, Tucson, AZ, United States

**Keywords:** insomnia, slow wave sleep (SWS), rapid eye movement (REM), continuous theta-burst stimulation (cTBS), repetitive transcranial magnetic stimulation (rTMS)

## Abstract

**Introduction:**

Insomnia is associated with mild cognitive impairment, although the mechanisms of this impairment are not well-understood. Timing of slow-wave and rapid eye movement sleep may help explain cognitive impairments common in insomnia. This investigation aimed to determine whether cognitive changes following continuous theta-burst stimulation (cTBS) are attributable to active stimulation, polysomnographic parameters of sleep, or both.

**Method:**

Data presented here are part of a pilot clinical trial aiming to treat insomnia by targeting a node in the default mode network using an inhibitory 40-s (cTBS). A double-blind counterbalanced sham-controlled crossover design was conducted. Participants (*N* = 20) served as their own controls on two separate in-laboratory visits—one with active cTBS and the other with sham cTBS. Each visit included cognitive assessments before and after stimulation and following a night of sleep in the lab monitored with polysomnography.

**Results:**

Slow wave sleep duration influenced working memory in the active cTBS condition, with shorter duration predicting improvements in working memory post sleep (*B* = −0.003, *p* = 0.095). Onset latency to rapid eye movement sleep predicted subsequent working memory, regardless of treatment condition (*B* = −0.001, *p* = 0.040). Results suggest that changes in attention and processing speed were primarily due to slow wave sleep onset (*B* = −0.001, *p* = 0.017) and marginally predicted by slow wave sleep duration (*B* = 0.002, *p* = 0.081) and sleep efficiency (*B* = 0.006, *p* = 0.090).

**Conclusions:**

Findings emphasize the important role that timing of slow-wave and rapid eye movement sleep have on information processing. Future work using larger sample sizes and more stimulation sessions is needed to determine optimal interactions between timing and duration of slow wave and rapid eye movement throughout the sleep period.

**Clinical trial registration:**

This study is registered on ClinicalTrials.gov (NCT04953559). https://clinicaltrials.gov/study/NCT04953559?locStr=Arizona&country=United%20States&state=Arizona&cond=insomnia&intr=tms%20&rank=1

## 1 Introduction

Difficulty falling asleep, maintaining a consistent sleep period, and daytime dysfunction related to these sleep disturbances are what characterize insomnia disorder (American Psychiatric Association, 2013). Regarding daytime dysfunction, insomnia is associated with mild cognitive impairment, although the mechanisms of this impairment are not well-understood (Wardle-Pinkston et al., 2019). One such mechanism that may contribute to the cognitive restoration associated with sleep is the organization of patterns of neural activity during sleep, such as slow wave oscillations and rapid eye movement (REM) duration (Van Someren et al., 2011). Indeed, slow wave sleep (SWS) and REM are associated with cognitive and emotional processing across human and animal studies (Straus et al., 2017; Walker and van der Helm, 2009; Silvestri, 2005; Silvestri and Root, 2008; Fu et al., 2007; Pace-Schott et al., 2009; Walker and Stickgold, 2004; Straus et al., 2018). For instance, impairments in attention have been associated with reduced SWS in individuals with insomnia (Li et al., 2016). Moreover, selective deprivation of REM sleep in mice results in significant impairment on learning and memory outcomes (Walker and Stickgold, 2004). A large cohort study in humans shows that greater cognitive impairment (i.e., global cognitive scores) is associated with less slow-wave and REM sleep stage duration (Haba-Rubio et al., 2017). Furthermore, individuals with chronic insomnia disorder (i.e., >1 year with symptoms) show decrements in cognitive performance, shorter SWS duration, and altered concentrations of blood-based neurodegenerative biomarkers (i.e., S100B, GFAP, GDNF, BDNF) compared with healthy controls (Zhang et al., 2020). Furthermore, older adults with insomnia who have less slow activity during sleep also display slower reaction times during cognitive testing compared to normal sleepers (Crenshaw and Edinger, 1999). Differences in resting-state functional connectivity within specific neural networks have also been associated with cognitive impairment in the chronic insomnia population (Pang et al., 2017).

Functional brain imaging in individuals with insomnia during resting state suggests that increased default mode network (DMN) activation and connectivity plays a role in the etiology and maintenance of insomnia symptoms because of its association with pre-sleep arousal and self-referential processing (Kay and Buysse, 2017). The DMN is known for its association with self-reflective thinking (e.g., daydreaming or introspection) while not engaged in any external tasks requiring attention (Mak et al., 2017). Prior findings suggest that in individuals with insomnia, DMN activity may reflect more of a ruminative resting state, associated with more persistent cognitive and physiological hyperarousal, thereby extending wake periods (Marques et al., 2018). Moreover, published findings from this trial suggest that the pre-sleep DMN activity correlates with multiple other cortical regions, the connectivity between which predicts sleep quality (Killgore W. D. et al., 2023). In individuals with insomnia, pre-sleep worry and physiological arousal contributes to delayed sleep onset as well as more awakenings and arousals during the sleep period (Kay and Buysse, 2017; Lancee et al., 2017). Prolonged elevations in arousal may deplete cognitive resources needed for tasks requiring attention. Cognitive functions such as attention, processing speed, and memory rely on multiple homeostatic and circadian processes regulating attention and alertness (Zhang and Gruber, 2019). Behavioral and pharmacological interventions for insomnia show that resultant changes in SWS commonly lead to subsequent changes on response-time attention tasks (Crenshaw and Edinger, 1999; Kim et al., 2013; Bazil et al., 2012). Data presented here are part of a pilot clinical trial addressing these cognitive and physiological aspects of arousal in insomnia by targeting the DMN using a brief, inhibitory 40-s continuous theta-burst stimulation (cTBS) of the left inferior parietal lobule, an external node in the dorsal DMN. This brief cTBS stimulation session was administered using repetitive transcranial magnetic stimulation (rTMS) and was aimed toward reducing overall excitability and connectivity across all regions comprising the DMN.

High-density electroencephalography studies indicate that slow wave oscillations originate in several of the brain regions that make up the DMN (i.e., medial prefrontal cortex, anterior cingulate cortex, precuneus, and posterior cingulate cortex) (Van Someren et al., 2011; Jerath and Crawford, 2015). According to the two-process model, the magnitude of slow wave oscillations is linked with sleep pressure built up from total time spent awake, as it is more homeostatically regulated than circadian alertness (Van Someren et al., 2011). Cognitive effects of SWS may rely on the timing of SWS onset in addition to duration (i.e., sleep depth). Earlier onset of SWS may lead to more slow wave oscillations expressed during the sleep window and potentially fewer slow waves expressed during wake (Van Someren et al., 2011). More slow wave oscillations during the day may inhibit cortical activation necessary to coordinate attention through increased sleep inertia (Ferrara et al., 2000). Data regarding SWS have been inconsistent, as some prior evidence shows greater time spent in SWS improves performance on various attention tasks whereas, others show the opposite effect (Ferrara et al., 2000; Matchock and Mordkoff, 2014; Diep et al., 2021). For example, some studies show that longer SWS stage durations are associated with decreased accuracy and reaction time on attention tasks (Ferrara et al., 2000; Matchock and Mordkoff, 2014). These findings may be more a product of sleep inertia and delayed timing of slow wave onset, due to prior sleep restriction.

Previously reported data from this trial indicate that active cTBS targeting the default mode network (DMN) shortens latency to SWS onset and may increase REM duration as compared to sham cTBS (Killgore W. et al., 2023). Similar studies using rTMS of non-DMN regions to treat insomnia have also found that several methods of rTMS protocols increase REM and SWS duration (Sun et al., 2021; Han et al., 2023); however, sleep staging has not been examined as mediating factors of cognitive improvement in these type of studies. We investigated whether differences in SWS onset timing and REM duration would predict greater improvements in cognitive performance in the active compared to sham conditions. The timing of REM onset is more regulated by light-regulated circadian phase (Charles et al., 1980) and is not expected to phase shift as quickly as SWS.

The current study investigated whether cognitive changes following cTBS rTMS are attributable to the stimulation, polysomnographic (PSG) parameters of sleep, or both. We hypothesized that changes in attention would be associated with SWS onset latency and REM duration, particularly in the active condition. Moreover, sleep parameters frequently associated with insomnia severity—latency to persistent sleep, sleep efficiency, and wake after sleep onset (WASO)—were examined as predictors of cognitive performance following stimulation and one night of sleep (Edinger et al., 2013). Shorter latency to persistent sleep, higher sleep efficiency, and less WASO were expected to predict improved cognitive performance.

## 2 Method

### 2.1 Participants and procedure

Twenty English speaking, relatively healthy adults (12 women; average age = 26.9, SD = 6.6 years) with self-reported sleep disturbances, insomnia symptoms, and/or daytime sleepiness were recruited from a southwestern community near a college campus. The absence of serious medical conditions, including other sleep disorders, were screened through an online survey. For breathing-related sleep disorders, including sleep apnea, the STOP-BANG was administered with scores over 3 or higher being ruled out (Chung et al., 2016). Participants also completed a general health questionnaire with multiple yes/no questions about prior diagnoses, including restless leg syndrome, sleep apnea, and recent travel outside of the local time zone. Individuals who reported having traveled outside of the local time zone were scheduled to a later visit date or excluded. Multiple neurological conditions potentially causing sleep disturbance were also screened out using the general health questionnaire as well (i.e., heart murmur, stroke, brain tumor, multiple sclerosis, amnesia, hydrocephalus). The general health questionnaire is a lab-designed instrument and not a validated questionnaire. Individuals who showed evidence of non-apnea sleep problems by scoring at or above conventional cutoffs for at least two of three established sleep problem scales were recruited [i.e., scored greater than or equal to six on the Pittsburgh Sleep Quality Index (PSQI), ≥15 on the Insomnia Severity Index (ISI), and/or ≥11 on the Epworth Sleepiness Scale (ESS)] (Buysse et al., 1989; Bastien et al., 2001; Johns, 1991).

Participants completed a double-blind counterbalanced sham-controlled crossover design in which they served as their own controls on two separate in-laboratory visits—one with active cTBS and the other with sham cTBS. Each visit included cognitive assessments before and after stimulation and following a night of sleep in the lab. Participants were randomly assigned to which treatment condition they would receive first. The randomization procedure was conducted using an allocation table to assure counterbalancing of treatment condition order. All study procedures were approved by a university Institutional Review Board as well as the Department of Defense Office of Human Research Oversight. See the published protocol for a detailed report of additional assessments beyond the scope of the current investigation (Hildebrand et al., 2024). This study is registered on ClinicalTrials.gov (NCT04953559) https://clinicaltrials.gov/study/NCT04953559?locStr=Arizona&country=United%20States&state=Arizona&cond=insomnia&intr=tms%20&rank=1.

**Figure 1:**
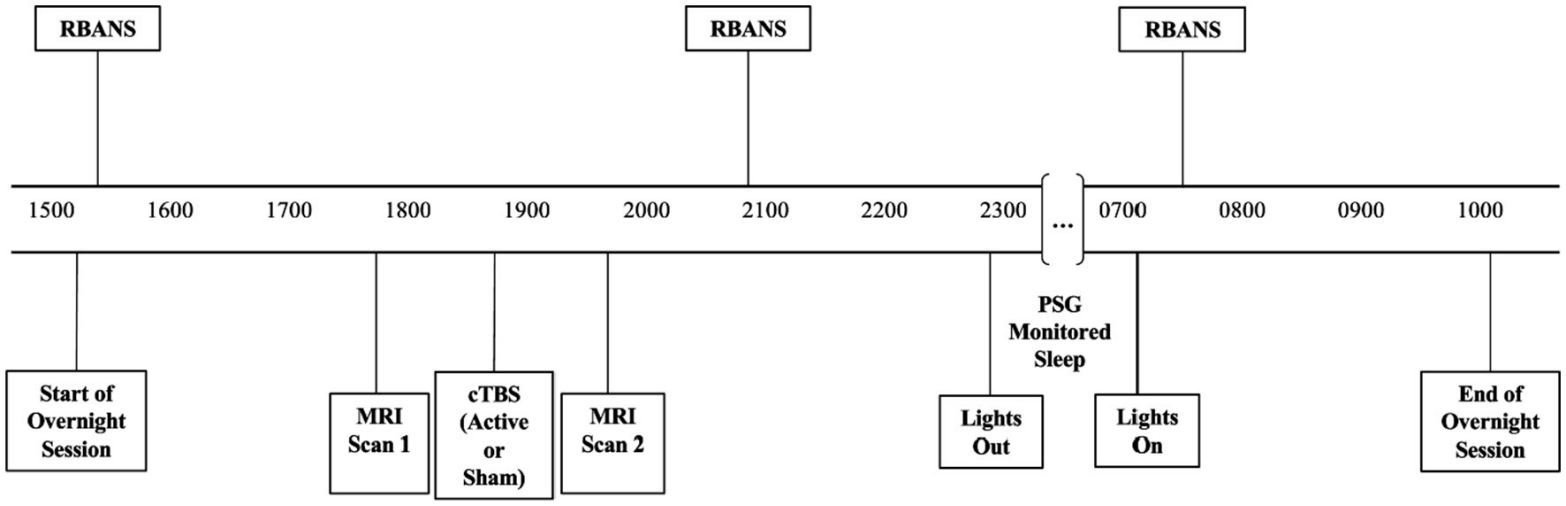
Timeline of cognitive assessments, stimulation, and polysomnographically monitored sleep conducted during both the active and sham conditions.

Sessions for both conditions included cognitive assessments conducted at baseline, after stimulation (active or sham) before bed, and the following morning (see Figure 1 for timeline). Although there were multiple cognitive assessments conducted pre-stimulation and following sleep, the Repeatable Battery for the Assessment of Neuropsychological Status (RBANS) was the only cognitive battery conducted before and after stimulation as well as post-sleep and was selected for current analysis for this reason. RBANS assessments at these three time points during each visit allowed us to differentiate whether changes in cognitive performance were due to treatment condition alone or whether PSG sleep parameters contributed to such changes.

### 2.2 Intervention: continuous theta-burst transcranial magnetic stimulation

Participants underwent the stimulation procedure in the evening at ~2000 hours, following a series of magnetic resonance imaging (MRI) scans. To maintain the double-blind component of the study design, the coil was prepared by research staff who were not present during stimulation. The coil was prepared in accordance with the participant's pre-assigned treatment condition for that visit—active cTBS or sham cTBS. The MagVenture Cool-B65 stimulator was connected to a figure-eight coil used for stimulation, which has an active side and a sham side. Stimulation intensity was set to 70% of an individual's resting motor threshold. Stimulation was applied to the IPL for 40 s a single time each visit. The T1-weighted image (i.e., Magnetization Prepared Rapid Gradient Echo) and the TMS 3D Neuronavigation system were used to localize the inferior parietal lobe (IPL)—this region of the DMN was selected due to the ease of access comparative to other regions within this network. The IPL was also chosen because a similar region of the parietal cortex was used in a previously tested TMS protocol and was shown to ameliorate insomnia and anxiety symptoms (Huang et al., 2018).

### 2.3 Instruments and measures

#### 2.3.1 Polysomnography

After the stimulation session, participants were allowed an 8-h sleep opportunity from 2300 to 0700 hours monitored with PSG, using the standard 10–20 electrode placement. Seven parameters were derived from PSG, including sleep stage onset latency and duration of N3 and REM, onset latency to persistent sleep (OLPS), sleep efficiency, and wake after persistent sleep onset (WAPSO). PSG files were scored by a registered polysomnographic technologist using the American Academy of Sleep Medicine scoring manual (Berry et al., 2017). Onset latency, duration, and WAPSO values are in minutes, whereas sleep efficiency is a percentage of total sleep time to total time in bed (i.e., how much they were asleep during the 8-h window). To determine whether participants' habitual sleep prior to lab visits differed markedly from the sleep opportunity provided in the lab, sleep-wake data from the Actiwatch Spectrum were examined regarding average bedtimes and waketimes (Respironics, 2005). On average, participants' at-home bedtime was 1 h and 19 min later than the laboratory 2300 bedtime and average at-home waketime was 23 min later than the laboratory 0700 waketime. Due to technical difficulties with the Actiwatch during screening days and nights, these data were only available from 18 out of the 20 total participants.

#### 2.3.2 Modified repeatable battery for the assessment of neuropsychological status (RBANS)

Versions of the digit-span forward, symbol coding, and story recall subtests of the RBANS exam were administered at all three timepoints (Randolph et al., 1998). The RBANS timepoints of one overnight visit are as follows: (1) baseline (T1), (2) post-stimulation (T2), and (3) post-sleep (T3).

##### 2.3.2.1 Digit span

Digit span forward is a measure of simple attention and working memory. For this task, a series of single-digit numbers were read aloud at a pace of one second apart and participants were asked to recall the numbers in sequence. Participants repeated a different set of digit strings at each of the three assessment timepoints during each visit and alternate forms were counterbalanced across visits.

##### 2.3.2.2 Symbol coding

Symbol coding captures attention, processing speed and learning. Participants were given a worksheet with a list of numbers and their associated symbols. After being given a chance to practice, participants are timed and decode as many numbers as possible for 90 s. Here we also assessed learning of the symbol-digit pairs across the visit by using the same assessment at each of the three timepoints in the same visit. Alternate forms were used across visits.

##### 2.3.2.3 Story recall

Story recall is a measure of verbal processing and auditory memory. For story recall, a single story was read to the participant, and they were asked to recall as many details as they could remember at each administration. For the baseline assessment of story recall, the story was read twice to the participant, then they were asked to provide immediate recall of story details on both administrations—the second administration was used as the baseline assessment used in the following analyses. After the baseline, the story was never read again. At the post-stimulation and post-sleep administrations, the participant was asked to recall the story in as much detail as possible to assess retention after the rTMS procedure and after a night of sleep. A new story was administered for a participant's second visit.

##### 2.3.2.4 Baseline-corrected change scores

For moderation models, baseline corrected change scores were created for each cognitive performance endpoint to capture the change from post-stimulation to post-sleep. To baseline correct values at each time point, post-stimulation and post-sleep values were each divided by baseline values (pre-cTBS). Values of post-stimulation performance were subtracted from values the following morning, so that increases during this period would become positive values and decreases, negative values. If T equals timepoint, then the change score calculation is: (T3/T1) – (T2/T1).

### 2.4 Analysis plan

To determine whether active cTBS stimulation impacts cognitive function immediately following stimulation and following an 8-h sleep opportunity, multilevel modeling was implemented using the R package *lmer* (Bates et al., 2014). A Kenward-Roger adjustment was implemented to approximate degrees of freedom and adjust the estimated standard deviations of the fixed effects using the *pbkrtest* package (Kenward and Roger, 1997; Halekoh and Højsgaard, 2014). A random intercept per individual was included in each model to account for the likely correlation among data points from the same individual. A linear mixed effects model with fixed effects for timepoint (i.e., post-stimulation and post-sleep) and treatment condition was fit to each cognitive performance variable (i.e., digit span, symbol coding, and story recall). To examine whether cognitive performance changed over time differently during sham vs. active lab visits, the interaction between timepoint and treatment condition was included. Participant sex and age were also added as fixed effects to determine whether these demographic factors influence cognitive performance. Random intercepts by individual were added to each model to account for individual person effects that are inherent in this counterbalanced RCT design in which participants were their own control subject. Contrasts between timepoints and treatment conditions were examined using the *emmeans* package (Lenth et al., 2018), which provides estimated marginal means of planned contrasts between effects derived from the mixed-effects models described above using the *lmer* package. Specifically, the contrasts examined for each cognitive variable included (1) comparing baseline to post-stimulation in the active and sham conditions, separately, (2) baseline to post-stimulation difference between active vs. sham, (3) baseline to post sleep in the active and sham conditions, separately, (4) baseline to post sleep difference between active and sham conditions, (5) post-stimulation to post sleep in the active and sham conditions, separately, (6) post-stimulation to post sleep difference between active vs. sham conditions. Data and model estimates are unstandardized.


$$\begin{array}{l} Y_{cognitive_{ij}}=\;\propto \;+\beta_{age}age_{ij}+\beta_{sex}\;I\;(sex_{ij}=\;female)\\ \;+\beta_{post-stim}\;I\;(time_{ij}=\;T2)+\;\beta_{post-sleep}\;I\;(time_{ij}=\;T3)\\ \;+\;\beta_{active}\;I\;(condition_{ij}=\;active)+\;\beta_{active\;\times \;post-stim}\\ \;I\;(time_{ij}=\;T2)\;I\;(condition_{ij}=\;active)+\\ \;\beta_{active\;\times \;post-sleep}\;I\;(time_{ij}=\;T3)\\ \;I\;(condition_{ij}=\;active)+\;\gamma_{i}+\;\varepsilon_{ij} \end{array}$$

Next, to determine whether objective sleep parameters affect cognitive performance, with or without active cTBS, moderation analyses were conducted also using *lmer* and *pbkrtest*. Linear mixed effects models with fixed effects for treatment condition, PSG variables of interest (i.e., SWS onset latency, SWS duration, REM onset latency, REM duration, OLPS, sleep efficiency, and WAPSO; see Supplementary Tables 1–3), and the interactions between PSG variables and treatment condition were fit for each cognitive performance pre- to post-sleep change score (i.e., digit span, symbol coding, and story recall). Models were fit separately for each PSG variable by treatment condition to examine specific impacts of each sleep stage of interest. Models with a PSG effect, but no interactive effect, were run without moderation to examine PSG effect across conditions. Model estimates are based on unstandardized data.


$$\begin{array}{l} Y_{cognitive\;change_{ij}}=\;\propto \;+\;\beta_{active}I\;\left(condition_{ij}=active\right)\;\\ \;+\beta_{PSG}PSG_{ij}+\;\beta_{active\;\times \;PSG}I\;\left(condition_{ij}=active\right)\\ \;PSG_{ij}\;+\;\gamma_{i}+\;\varepsilon_{ij} \end{array}$$

All latency, stage duration, and WAPSO variables were expressed in minutes. Sleep efficiency calculations were based on a percentage of time spent asleep during the sleep window. Analyses presented here included PSG parameters calculated from the entire 8-h sleep window with one exception. Due to an unanticipated event, a participant was woken up 1 h early during their active condition visit. Therefore, the sleep efficiency calculation for this participant during their active visit was calculated based on a total of 7 h in bed instead of 8 h.

## 3 Results

### 3.1 Interactive effects of treatment condition and time of administration

**Figure 2:**
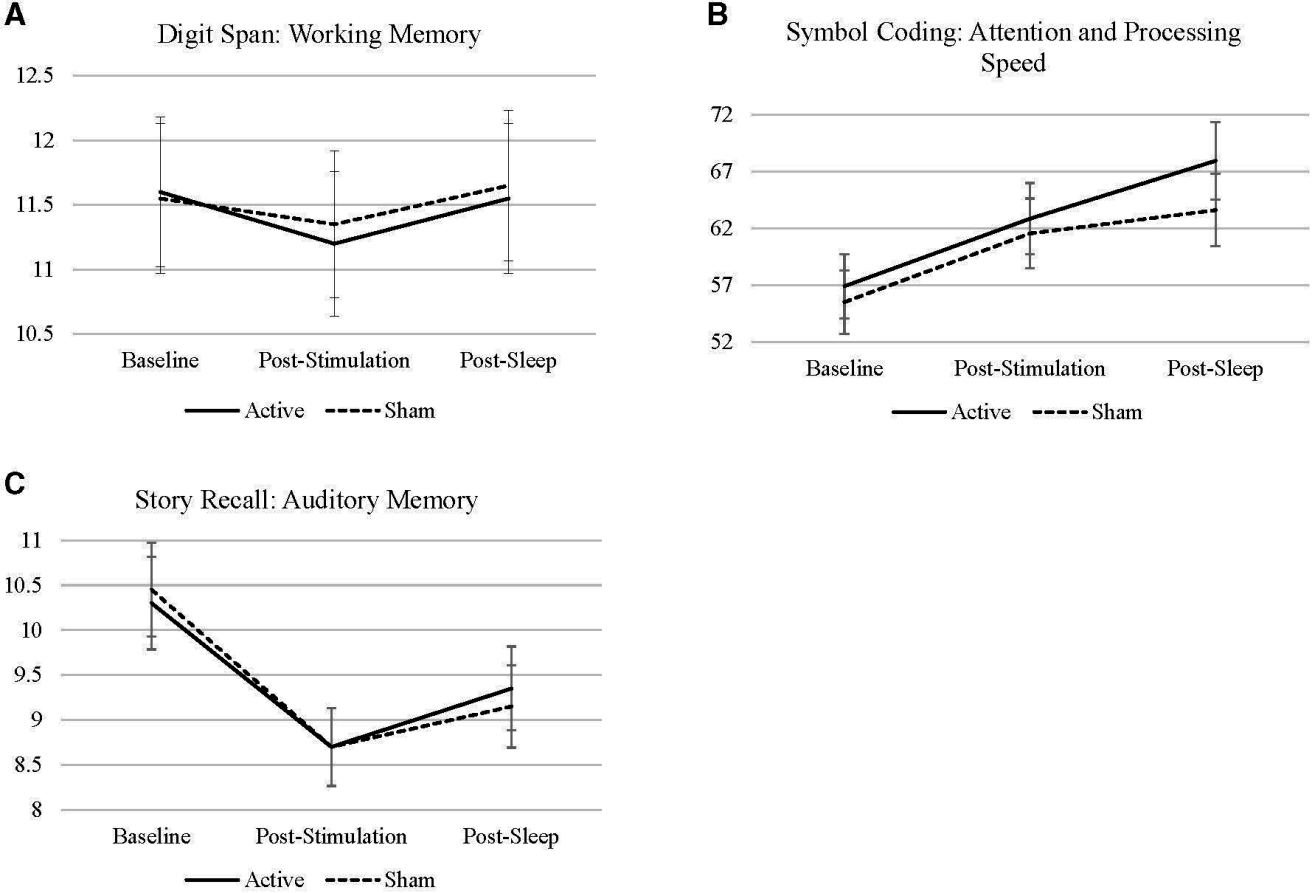
**(A–C)** Line charts represent the raw scores of digit span, symbol coding, story recall across all timepoints sample during each visit.

There were no significant interactions between treatment condition by time of administration (baseline to post-stimulation, *p* = 0.762; baseline to post-sleep, *p* = 0.820) on digit span performance. There were no main effects of treatment condition (i.e., active vs. sham; *p* = 0.915) or time of administration, regardless of treatment condition (*p* = 0.669; *p* = 0.830), on digit span performance. Sex (*p* = 0.141) and age (*p* = 0.801) did not have a significant impact on digit span performance. Means and standard deviations by treatment condition are presented in Figure 2.

There were no interactions between treatment condition from baseline to post-stimulation (*p* = 0.518) or from baseline to post-sleep (*p* = 0.336) on coding performance. There was a main effect of time on coding performance, such that performance improved from baseline to immediately post-stimulation (*EM* = 6.00, *S.E*. = 1.53, *p* < 0.001) and following sleep (*EM* = 9.57, *S.E*. = 1.53, *p* < 0.001). Treatment condition did not have a main effect on overall coding performance (*p* = 0.518). Immediate effects of stimulation (i.e., baseline to post-stimulation) on coding performance were significant in both the active (*EM* = 5.95, *S.E*. = 2.16, *p* = 0.007) and sham conditions (*EM* = 6.05, *S.E*. = 2.16, *p* = 0.006), but this effect did not differ between treatment conditions (*p* = 0.934). The effect of stimulation and a night of sleep on coding performance (i.e., baseline to post sleep scores) was numerically greater during the active condition (*EM* = 11.05, *S.E*. = 2.16, *p* < 0.001) compared to sham (*EM* = 8.10, *S.E*. = 2.16, *p* < 0.001), but the effects did not differ statistically significantly between conditions *(p* = 0.336). The comparison from post-stimulation to post-sleep was significant in the active condition (*EM* = 5.10, *S.E*. = 2.16, *p* = 0.020), but not in the sham condition (*p* = 0.320)—these effects were not statistically different by treatment condition (*p* = 0.320). See Table 1 for all contrasts. This suggests that the benefits of cTBS on attention and processing speed may be conferred by specific sleep characteristics impacted by active cTBS—this is explored further in the moderation analyses below.

**Table 1:** Estimated marginal means of cognitive variables over time and by treatment condition.

	**EM**	***SE***	***df***	***t***	***p***
**Digit-span contrasts**					
Baseline to post-stim: sham	−0.200	0.466	95	−0.429	0.669
Baseline to post-stim: active	−0.400	0.466	95	−0.859	0.393
Baseline to post-stim: active—sham	−0.200	0.659	95	−0.304	0.762
Baseline to post-stimulation: averaged	−0.300	0.329	95	−0.911	0.365
Baseline to post-sleep: sham	0.100	0.466	95	0.215	0.830
Baseline to post-sleep: active	−0.050	0.466	95	−0.107	0.915
Baseline to post-sleep: active—sham	−0.150	0.659	95	−0.228	0.820
Post-stimulation to post-sleep: averaged	0.025	0.329	95	0.076	0.940
Post-stim to post-sleep: sham	0.300	0.466	95	0.644	0.521
Post-stim to post-sleep: active	0.350	0.466	95	0.752	0.454
Post-stim to post-sleep: active—sham	0.050	0.659	95	0.076	0.940
**Symbol-coding contrasts**					
Baseline to post-stim: sham	6.050	2.160	95	2.803	0.006
Baseline to post-stim: active	5.950	2.160	95	2.757	0.007
Baseline to post-stim: active—sham	−0.100	3.050	95	−0.033	0.974
Baseline to post-stimulation: averaged	6.000	1.530	95	3.932	<0.0001
Baseline to post-sleep: sham	8.100	2.160	95	3.753	<0.0001
Baseline to post-sleep: active	11.050	2.160	95	5.120	<0.0001
Baseline to post-sleep: active—sham	2.950	3.050	95	0.967	0.336
Post-stimulation to post-sleep: averaged	9.570	1.530	95	6.274	<0.0001
Post-stim to post-sleep: sham	2.050	2.160	95	0.950	0.345
Post-stim to post-sleep: active	5.100	2.160	95	2.363	0.020
Post-stim to post-sleep: active—sham	3.050	3.050	95	0.999	0.320
**Story recall contrasts**					
Baseline to post-stim: sham	−1.750	0.365	95	−4.795	<0.0001
Baseline to post-stim: active	−1.600	0.365	95	−4.384	<0.0001
Baseline to post-stim: active—sham	0.150	0.516	95	0.291	0.772
Baseline to post-stimulation: averaged	−1.68	0.258	95	−6.490	<0.0001
Baseline to post-sleep: sham	−1.300	0.365	95	−3.562	0.001
Baseline to post-sleep: active	−0.950	0.365	95	−2.603	0.011
Baseline to post-sleep: active—sham	0.350	0.516	95	0.678	0.499
Post-stimulation to post-sleep: averaged	−1.120	0.258	95	−4.359	<0.0001
Post-stim to post-sleep: sham	0.450	0.365	95	1.233	0.221
Post-stim to post-sleep: active	0.650	0.365	95	1.781	0.078
Post-stim to post-sleep: active—sham	0.200	0.516	95	0.387	0.699

Age influenced overall coding performance [*B* = −0.887, *t*_(7)_ = −2.127, *p* = 0.048], such that older participants had lower scores on average. To further examine the effects of age at each timepoint in both conditions, bivariate correlations were run, separately by treatment condition. When examined by timepoint and treatment condition, age predicted coding performance in the post-sleep timepoint only [*B* = −0.8166, *t*_(90)_ = −2.523, *p* = 0.013] and was not moderated by treatment condition.

There were no interactions between treatment condition from baseline to post-stimulation (*p* = 0.772) or from baseline to post-sleep (*p* = 0.499) on story recall. There was an effect of time of administration on story recall, such that recall decreased from baseline to post-stimulation (*EM* = −1.68, *SE* = 0.258, *p* < 0.001) and from baseline to post-sleep (*EM* = −1.12, *SE* = 0.258, *p* < 0.001). There was no main effect of treatment condition on story recall (*p* = 0.682). There was a numerically smaller decline in story recall from baseline to post-stimulation in the active condition (*EM* = −1.60, *S.E*. = 0.365, *p* < 0.001) compared to sham (*EM* = −1.75, *S.E*. = 0.365, *p* < 0.001); although, these contrasts were not significantly different from each other (*p* = 0.772). Similarly, there was less decline in story recall from baseline to post-sleep in the active condition (*EM* = −0.95, *S.E*. = 0.365, *p* = 0.011) compared to sham (*EM* = −1.30, *S.E*. = 0.365, *p* < 0.001); these effects were not statistically significantly different (*p* = 0.499). Changes in story recall from post-stimulation (i.e., pre-sleep) to post sleep became positive and, in the active condition only, although this effect is marginal (*EM* = 0.65, *S.E*. = 0.365, *p* = 0.078).

### 3.2 Effects of polysomnographic sleep and treatment condition

**Figure 3:**
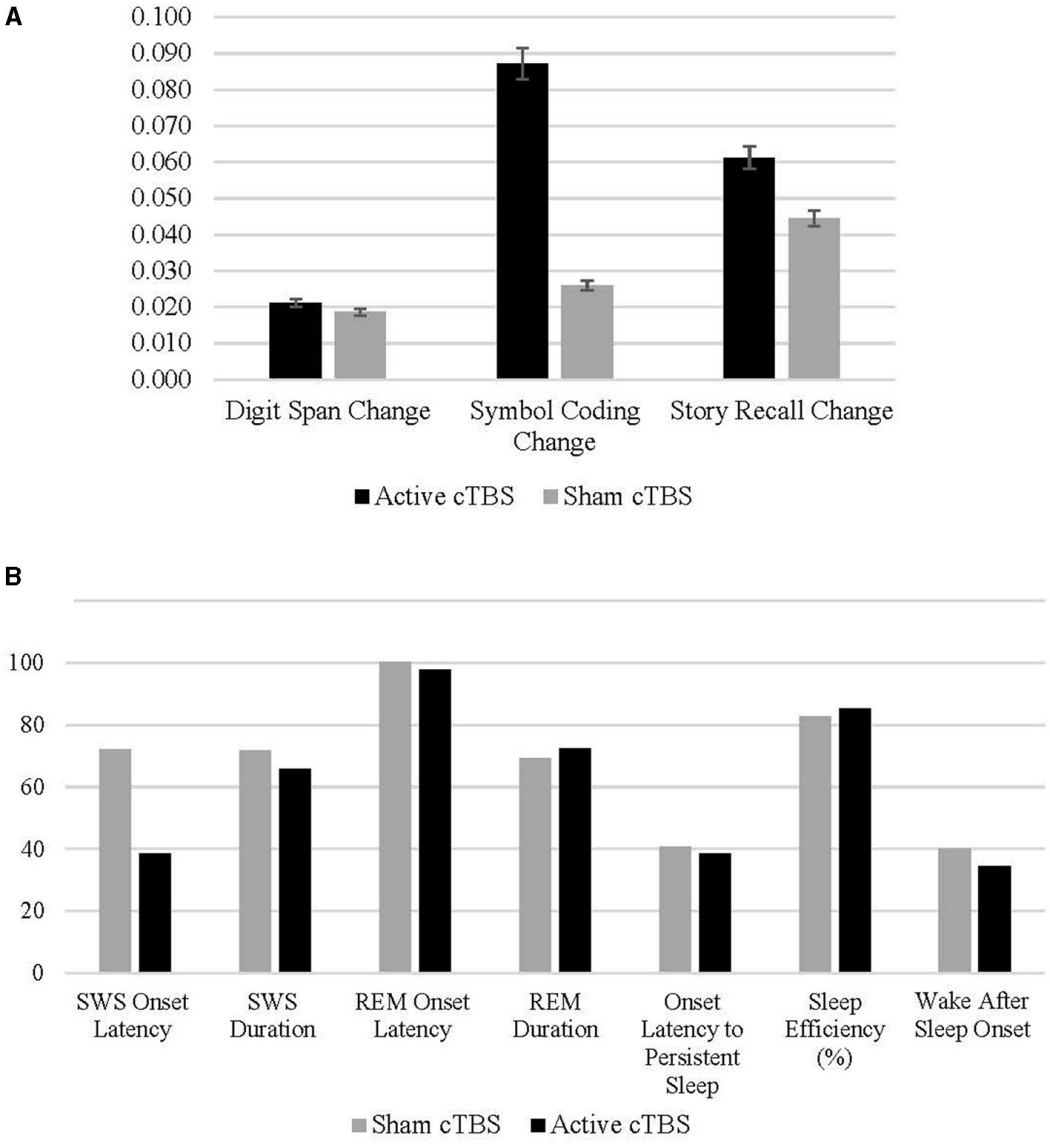
**(A, B)** Means and standard deviations of change in cognitive performance pre- to post-sleep in the active and sham conditions.

To examine the change in cognitive performance following an 8-h sleep window more closely, change scores for digit span, symbol coding, and story recall were used as the outcome criterion variables in the following moderation models (see Section 2.3.2.4 above for change score calculations). See Figure 3 for cognitive change scores and PSG parameters by treatment condition.

**Figure 4:**
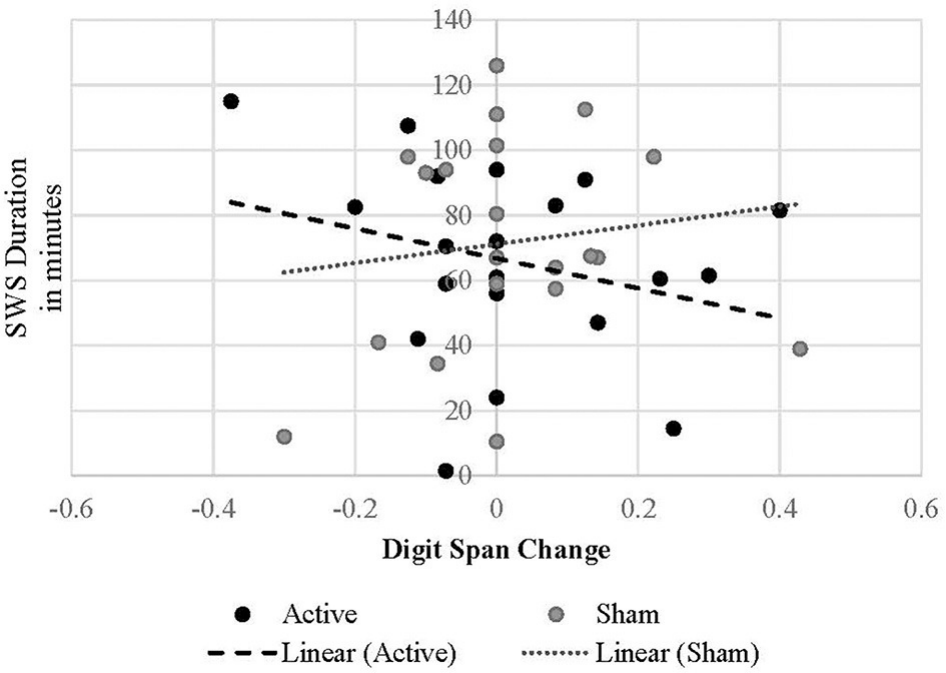
Linear relationship between SWS duration and digit span performance change in each treatment condition.

Regarding change in digit span performance from post-stimulation to post-sleep, there were not statistically significant (*p* < 0.05) interactive effects of treatment condition by any PSG variable on cognitive change. However, the interaction between treatment condition and SWS duration was marginal [*B* = −0.003, *t*_(19.438)_ = −1.753, *p* = 0.095], suggesting that in the active treatment condition longer SWS duration may predict a decrease in digit span performance following active cTBS and a night of sleep (Figure 4). There were no effects of REM onset latency unique to the active condition (*p* = 0.347). However, in both conditions, shorter REM onset latency was associated with improved digit-span performance after sleep [*B* = −0.001, *t*_(37.794)_ = −2.180, *p* = 0.040].

**Figure 5:**
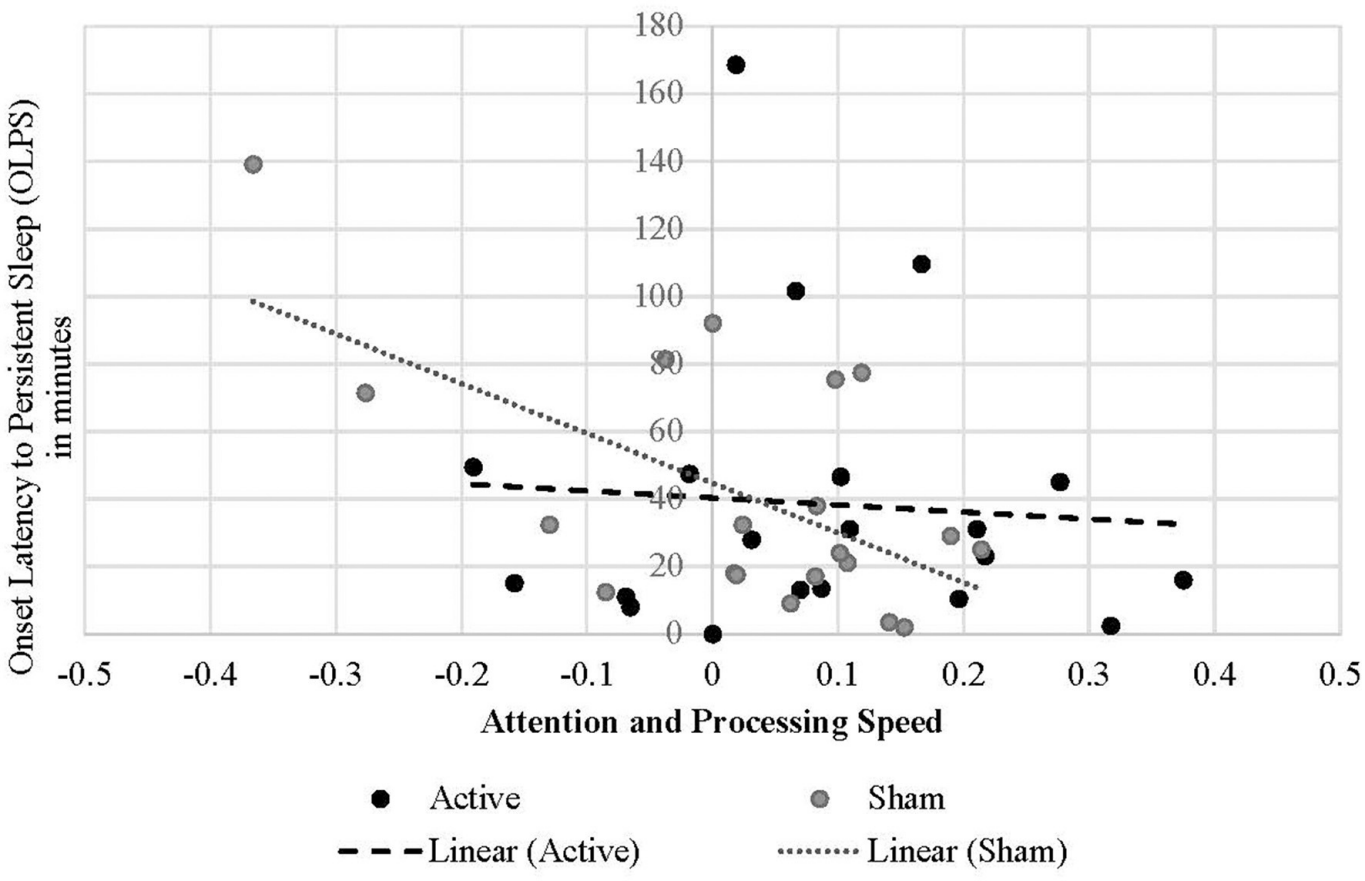
Linear relationship between onset latency to persistent sleep and attention and processing speed performance change in each treatment condition.

Treatment condition moderated the relationship between OLPS and symbol coding performance, such that shorter OLPS predicted greater change in coding scores during the sham condition [*B* = −0.002, *t*_(34.032)_ = −2.464, *p* = 0.019; Figure 5; Table 2]. However, the effect of OLPS on coding in the active condition was equal to zero [calculated estimate for active group is 0.002 + (−0.002) = 0]. In the sham condition only, there was a marginal positive effect of SWS duration [*B* = 0.002, *t*_(34.764)_ = 1.797, *p* = 0.081] on increased coding scores that trended toward significance. There was no interactive effect of active condition (*p* = 0.494) by SWS duration on coding. Across both conditions, coding performance was also influenced by SWS onset latency [*B* = −0.001, *t*_(28.730)_ = −2.542, *p* = 0.017], such that shorter onset to SWS predicted greater improvement in coding performance. Additionally, higher sleep efficiency percentage predicted improvements in coding performance [*B* = 0.006, *t*_(34.399)_ = 1.743, *p* = 0.090] in both active and sham conditions.

**Table 2:** Means and standard deviations of cognitive change scores and polysomnography parameters by treatment condition.

		**Active cTBS**	**Sham cTBS**
**Cognitive change scores**			
Digit span change	*M*	0.02	0.02
	*SD*	0.18	0.15
Symbol coding change	*M*	0.09	0.03
	*SD*	0.15	0.15
Story recall change	*M*	0.06	0.04
	*SD*	0.12	0.12
**Polysomnography parameters**			
SWS onset latency	*M*	38.53	72.28
	*SD*	42.47	64.99
SWS duration	*M*	65.80	71.68
	*SD*	29.74	32.96
REM onset latency	*M*	97.78	100.20
	*SD*	50.86	53.54
REM duration	*M*	72.58	69.28
	*SD*	34.39	25.85
Sleep onset latency to persistent sleep	*M*	38.53	40.93
	*SD*	42.47	36.25
Sleep efficiency (%)	*M*	85.30	84.36
	*SD*	10.62	10.42
Wake after sleep onset	*M*	34.60	40.10
	*SD*	34.55	36.21

PSG variables are in minutes except for sleep efficiency, which is in percent.

Because age was a significant predictor of coding in the main effects growth models, age was added as a predictor in the symbol coding performance moderation models to account for its influence. However, there were no significant effects of age on coding performance change scores.

The change in story recall performance was not influenced by treatment condition or sleep staging, nor the interactions between them.

## 4 Discussion

This investigation aimed to determine whether cognitive changes following cTBS are attributable to active stimulation, PSG parameters of sleep, or both. Overall, cTBS did not appear to directly affect attention and working memory, learning and processing speed, or retention of verbal information. Results suggest that changes in attention and processing speed were due more to objective sleep quality than acute changes from cTBS. Changes in working memory were partially due to PSG parameters of sleep. Longitudinal models of cognitive performance across each visit show that, from baseline to post-stimulation and post-sleep, cognitive performance did not differ statistically between treatment conditions. However, attention and processing speed (i.e., symbol coding) performance and verbal-auditory memory (i.e., story recall) improved following active cTBS stimulation, plus a night of sleep. Age was also found to impact attention and processing speed performance, such that younger participants performed better on this task, overall, particularly following a night of sleep. There were no interactive treatment condition effects regarding verbal-auditory recall. The decrease in verbal-auditory recall from baseline was anticipated, due to the increasing length of time from initially hearing the story to post-stimulation and then post-sleep.

### 4.1 Interactive effect of objective sleep quality

Shorter SWS duration predicted working memory improvements in the active condition. Treatment condition also moderated latency to persistent sleep, such that shorter latency to persistent sleep was associated with improved attention and processing speed in the sham condition only. Following a night of sleep, story recall increased significantly in the active condition. However, story recall was not affected by treatment condition or sleep parameters, nor any interactions between them.

Previous studies in which rTMS was implemented to improve insomnia symptoms show that increasing SWS duration may be a mechanism contributing to improved sleep quality and insomnia symptoms, including self-reported improvements (Sun et al., 2021; Jiang et al., 2019a). However, prior evidence and findings presented here show mixed effects of SWS duration regarding performance on attention and working memory tasks (Ferrara et al., 2000; Matchock and Mordkoff, 2014; Diep et al., 2021; Ferrarelli et al., 2019). Individuals with insomnia tend to have less slow wave activity during the first part of the night, which leads to a slower dissipation of slow waves over the remainder of the night and worsened performance on complex attention task in the morning. Younger vs. older individuals have most SWS in the earlier half of the sleep window (Hayashi and Endo, 1982). In the active condition, lower SWS duration was associated with better working memory. This may be more due to the timing of slow wave sleep on waking cognitive capacity than the duration. Previously published findings from this study indicate that SWS onsets earlier in the active condition compared to sham (Killgore W. et al., 2023). It is likely that earlier onset SWS in the active cTBS condition led to shorter duration on this night, which enhanced working memory performance the following morning. Additionally, we found that earlier SWS onset and longer SWS duration predict improved attention and processing speed across both active and sham conditions. Earlier onset of SWS reduces the likelihood of slow wave expression near or into the early waking period, which is associated with greater sleep inertia following the sleep period (Van Someren et al., 2011). Treatments that advance the timing of SWS onset may increase the likelihood of more consolidated slow waves earlier in the night—further away from the waking window—thereby improving working memory and complex attention (Lunsford-Avery et al., 2022). It is important to note that slow wave activity (SWA) and the N3 stage classifications are not the same metric, although they reflect similar sleep parameters, with one major difference being that SWA can be detected in both sleep and wake states.

Similarly, shorter onset to REM sleep predicted improved working memory across conditions. Shorter REM onset latency is associated with insomnia and other psychiatric conditions (Omichi et al., 2022; Riemann, 2007). Although REM duration has been linked with working memory enhancement, REM latency has not been. To speculate, it is possible that individuals with an earlier REM onset also had longer REM durations, thereby indirectly affecting working memory.

Regarding the insomnia-related PSG variables (Edinger et al., 2013), sleep efficiency is the only one that had an effect on cognitive performance. Greater sleep efficiency predicted improved attention and processing speed across both active and sham conditions. Sleep efficiency is a common measure of sleep quality, as it captures the amount of time in bed that one spent asleep. The overall effect of sleep efficiency improving attention and processing speed is supported by prior findings (Miyata et al., 2013).

### 4.2 Importance of the placebo effect in sham comparison designs

The interpretation of sham effects on cognitive performance being more attributable to sleep may not be the full picture, as there is evidence that TMS has placebo effects across multiple clinical samples, including insomnia (Jiang et al., 2019a; Razza et al., 2018). For instance, a meta-analysis of rTMS interventions used to treat insomnia disorder showed that 73% of the treatment effect in active groups could be attributed to a placebo effect (Jiang et al., 2019a). In the present investigation, *post-hoc* correlations reveal that in the sham condition alone, shorter latency to SWS and persistent sleep, longer SWS duration, and higher sleep efficiency were correlated with improved attention and processing speed after sleep. Specifically, in studies using rTMS to improve sleep, the effect of sham is highly significant, particularly regarding SWS and REM duration, latency to persistent sleep, and sleep efficiency (Jiang et al., 2019a). The placebo effect has also been shown to improve insomnia symptoms in response to rTMS (Jiang et al., 2019b).

### 4.3 Limitations

This clinical trial was a proof-of-concept pilot study examining whether targeting an outer node of the DMN with inhibitory rTMS would improve polysomnographically measured sleep, thus only 20 participants were recruited. This small sample poses some limitations for statistical power in detecting measurable outcomes associated with stimulation. Secondly, while insomnia symptoms are a focus of the study, our screening approach was broad and simply required individuals to score above standard cutoffs for at least two of three scales assessing general sleep disturbance issues (i.e., PSQI), insomnia severity (i.e., ISI), and/or daytime sleepiness (i.e., ESS). This approach may have increased the variability in the types of sleep issues that were included in the study. Future research should focus specifically on more well-defined populations such as those meeting criteria for insomnia disorder. Additionally, because this study was a pilot investigation, only a single 40 s stimulation train was administered as the intervention. The number of stimulation sessions and their duration have meaningful effects on changing neural activity and associated outcomes of rTMS, including sleep quality and insomnia symptoms (Jiang et al., 2019a; Oroz et al., 2021). Previous investigations of rTMS as a treatment for insomnia suggest a clear dose-response association such that more stimulation sessions across 30 days have greater effects on insomnia than fewer sessions (Jiang et al., 2019a). A subsequent clinical trial is needed to evaluate the effectiveness of inhibitory cTBS to multiple nodes of the DMN across multiple sessions and days in treating individuals with insomnia.

Given that the focus of the pilot study was on polysomnographic outcomes, insomnia and sleep disturbance-related symptoms were not sampled again following sham and active treatment conditions. Future studies using rTMS to improve sleep quality and treat insomnia should include a larger sample, increase the stimulation sessions across multiple days, and assess insomnia systems and self-reported sleep quality following treatment.

Considering the effects that habitual sleep regularity has on sleep latency and slow wave sleep (Feinberg et al., 1987; Vital-Lopez et al., 2021; Aeschbach et al., 1996), it is important to note that some participants' sleep in the prior week was not consistent with the permitted laboratory sleep window. Specifically, participants' at-home bedtime was an average of 1 h and 19 min later than the 2300 hours laboratory bedtime and average at-home waketime was 23 min later than the 0700 hours laboratory waketime. Future studies examining these treatment effects should consider conducting in-home PSG as a treatment outcome marker to increase the ecological validity of sleep staging data (Sánchez-Ortuño et al., 2010).

## 5 Summary

Findings emphasize the important role that timing of slow-wave and rapid eye movement sleep have on information processing. Increasing early onset SWS as well as SWS and REM duration be an apt treatment target for improving insomnia-related cognitive impairment. The effect of active cTBS on SWS onset latency to influence cognitive performance may be detectible with a larger sample, particularly considering that previous findings—active inhibition of the DMN through cTBS significantly reduced onset latency to SWS (Killgore W. et al., 2023).

Future work using larger sample sizes and more stimulation sessions is needed to determine optimal interactions between timing and duration of slow wave and rapid eye movement throughout the sleep period. This method of cortical inhibition via cTBS holds promise for improving cognition and sleep quality in individuals with insomnia.

## Data Availability

The raw data supporting the conclusions of this article will be made available by the authors, without undue reservation.
