# Supplementary material for: Polysomnographically mediated cognitive improvements in individuals with insomnia symptoms following continuous theta-burst stimulation of the default mode network
Source: Front Sleep. 2024 Oct 2;3:1424083. doi: 10.3389/frsle.2024.1424083 (PMC12713820; doi:10.3389/frsle.2024.1424083)
Supplement: Supplementary file 1 [file Table_1.DOCX]

Supplementary Material

# Supplementary Tables

Supplementary Table 1.

Moderation models examining interactive effects between treatment condition and polysomnographic sleep parameters on digit-span performance change from post-stimulation to post-sleep.

| **Outcome: Digit-Span Change** | | |  | |  | |  | |  | |  | |  | |
| --- | --- | --- | --- | --- | --- | --- | --- | --- | --- | --- | --- | --- | --- | --- |
| Intercept | Est. | 0.085 | -0.033 | | 0.169 | | 0.069 | | 0.045 | | -0.230 | | 0.046 | |
|  | *CI* | (-0.031,0.200) | (-0.219,0.153) | | (0.0114,0.326) | | (-0.16,0.298) | | (-0.075,0.165) | | (-0.868,0.408) | | (-0.07,0.163) | |
|  | *df* | 34.759 | 35.337 | | 35.245 | | 35.961 | | 34.139 | | 35.680 | | 35.992 | |
|  | *t* | 1.484 | -0.356 | | 2.177 | | 0.612 | | 0.764 | | -0.732 | | 0.808 | |
|  | *p* | 0.147 | 0.724 | | 0.036 | | 0.544 | | 0.450 | | 0.469 | | 0.425 | |
|  |  |  |  | |  | |  | |  | |  | |  | |
| **Active cTBS (1)** | Est. | -0.023 | | 0.170 | | -0.081 | | -0.041 | | 0.000 | | 0.413 | | -0.059 |
|  | *CI* | (-0.171,0.126) | | (-0.050,0.390) | | (-0.274,0.120) | | (-0.306,0.224) | | (-0.137,0.137) | | (-0.363,1.189) | | (-0.198,0.080) |
|  | *df* | 19.188 | | 19.023 | | 20.989 | | 21.389 | | 18.7642 | | 19.374 | | 20.878 |
|  | *t* | -0.321 | | 1.6145 | | -0.877 | | -0.320 | | 0.001 | | 1.112 | | -0.880 |
|  | *p* | 0.752 | | 0.1229 | | 0.391 | | 0.752 | | 0.9995 | | 0.280 | | 0.389 |
|  |  |  | |  | |  | |  | |  | |  | |  |
| **SWS Latency** | Est. | -0.001 | |  | |  | |  | |  | |  | |  |
|  | *CI* | (-0.002,0.004) | |  | |  | |  | |  | |  | |  |
|  | *df* | 35.881 | |  | |  | |  | |  | |  | |  |
|  | *t* | -1.538 | |  | |  | |  | |  | |  | |  |
|  | *p* | 0.133 | |  | |  | |  | |  | |  | |  |
|  |  |  | |  | |  | |  | |  | |  | |  |
| **Active cTBS × SWS Latency** | Est. | 0.0002 | |  | |  | |  | |  | |  | |  |
|  | *CI* | (-0.002,0.002) | |  | |  | |  | |  | |  | |  |
|  | *df* | 22.531 | |  | |  | |  | |  | |  | |  |
|  | *t* | 0.166 | |  | |  | |  | |  | |  | |  |
|  | *p* | 0.870 | |  | |  | |  | |  | |  | |  |
|  |  |  | |  | |  | |  | |  | |  | |  |
| **SWS Duration** | Est. |  | | 0.001 | |  | |  | |  | |  | |  |
|  | *CI* |  | | (-0.002,0.003) | |  | |  | |  | |  | |  |
|  | *df* |  | | 35.7543 | |  | |  | |  | |  | |  |
|  | *t* |  | | 0.6135 | |  | |  | |  | |  | |  |
|  | *p* |  | | 0.5434 | |  | |  | |  | |  | |  |
|  |  |  | |  | |  | |  | |  | |  | |  |
| **Active cTBS × SWS Duration** | Est. |  | | -0.003 | |  | |  | |  | |  | |  |
|  | *CI* |  | | (-0.005,0.000) | |  | |  | |  | |  | |  |
|  | *df* |  | | 19.438 | |  | |  | |  | |  | |  |
|  | *t* |  | | -1.753 | |  | |  | |  | |  | |  |
|  | *p* |  | | 0.095 | |  | |  | |  | |  | |  |
|  |  |  | |  | |  | |  | |  | |  | |  |
| **REM Latency** | Est. |  | |  | | -0.002 | |  | |  | |  | |  |
|  | *CI* |  | |  | | (-0.003, 0.000) | |  | |  | |  | |  |
|  | *df* |  | |  | | 32.026 | |  | |  | |  | |  |
|  | *t* |  | |  | | -2.215 | |  | |  | |  | |  |
|  | *p* |  | |  | | 0.034 | |  | |  | |  | |  |
|  |  |  | |  | |  | |  | |  | |  | |  |
| **Active cTBS × REM Latency** | Est. |  | |  | | 0.001 | |  | |  | |  | |  |
|  | *CI* |  | |  | | (0.001,0.003) | |  | |  | |  | |  |
|  | *df* |  | |  | | 21.903 | |  | |  | |  | |  |
|  | *t* |  | |  | | 0.961 | |  | |  | |  | |  |
|  | *p* |  | |  | | 0.347 | |  | |  | |  | |  |
|  |  |  | |  | |  | |  | |  | |  | |  |
| **REM Duration** | Est. |  | |  | |  | | -0.001 | |  | |  | |  |
|  | *CI* |  | |  | |  | | (-0.004,0.002) | |  | |  | |  |
|  | *df* |  | |  | |  | | 35.763 | |  | |  | |  |
|  | *t* |  | |  | |  | | -0.476 | |  | |  | |  |
|  | *p* |  | |  | |  | | 0.637 | |  | |  | |  |
|  |  |  | |  | |  | |  | |  | |  | |  |
| **Active cTBS × REM Duration** | Est. |  | |  | |  | | 0.001 | |  | |  | |  |
|  | *CI* |  | |  | |  | | (-0.003,0.004) | |  | |  | |  |
|  | *df* |  | |  | |  | | 22.251 | |  | |  | |  |
|  | *t* |  | |  | |  | | 0.372 | |  | |  | |  |
|  | *p* |  | |  | |  | | 0.713 | |  | |  | |  |
|  |  |  | |  | |  | |  | |  | |  | |  |
| **Latency to Persistent Sleep** | Est. |  | |  | |  | |  | | -0.0006 | |  | |  |
|  | *CI* |  | |  | |  | |  | | (-0.003,0.002) | |  | |  |
|  | *df* |  | |  | |  | |  | | 35.090 | |  | |  |
|  | *t* |  | |  | |  | |  | | -0.591 | |  | |  |
|  | *p* |  | |  | |  | |  | | 0.558 | |  | |  |
|  |  |  | |  | |  | |  | |  | |  | |  |
| **Active cTBS x Latency to Persistent Sleep** | Est. |  | |  | |  | |  | | 0.000 | |  | |  |
|  | *CI* |  | |  | |  | |  | | (-0.003,0.003) | |  | |  |
|  | *df* |  | |  | |  | |  | | 19.328 | |  | |  |
|  | *t* |  | |  | |  | |  | | 0.021 | |  | |  |
|  | *p* |  | |  | |  | |  | | 0.984 | |  | |  |
|  |  |  | |  | |  | |  | |  | |  | |  |
| **Sleep Efficiency** | Est. |  | |  | |  | |  | |  | | 0.003 | |  |
|  | *CI* |  | |  | |  | |  | |  | | (-0.004,0.011) | |  |
|  | *df* |  | |  | |  | |  | |  | | 35.418 | |  |
|  | *t* |  | |  | |  | |  | |  | | 0.880 | |  |
|  | *p* |  | |  | |  | |  | |  | | 0.385 | |  |
|  |  |  | |  | |  | |  | |  | |  | |  |
| **Active cTBS x Sleep Efficiency** | Est. |  | |  | |  | |  | |  | | -0.004 | |  |
|  | *CI* |  | |  | |  | |  | |  | | (-0.014,0.005) | |  |
|  | *df* |  | |  | |  | |  | |  | | 19.177 | |  |
|  | *t* |  | |  | |  | |  | |  | | -0.937 | |  |
|  | *p* |  | |  | |  | |  | |  | | 0.360 | |  |
|  |  |  | |  | |  | |  | |  | |  | |  |
| **Wake After Persistent Sleep Onset** | Est. |  | |  | |  | |  | |  | |  | | -0.001 |
|  | *CI* |  | |  | |  | |  | |  | |  | | (-0.003,0.002) |
|  | *df* |  | |  | |  | |  | |  | |  | | 32.562 |
|  | *t* |  | |  | |  | |  | |  | |  | | -0.653 |
|  | *p* |  | |  | |  | |  | |  | |  | | 0.519 |
|  |  |  | |  | |  | |  | |  | |  | |  |
| **Active cTBS × Wake After Sleep Onset** | Est. |  | |  | |  | |  | |  | |  | | 0.002 |
|  | *CI* |  | |  | |  | |  | |  | |  | | (-0.001,0.005) |
|  | *df* |  | |  | |  | |  | |  | |  | | 23.737 |
|  | *t* |  | |  | |  | |  | |  | |  | | 1.198 |
|  | *p* |  | |  | |  | |  | |  | |  | | 0.243 |

Supplementary Table 2.

Moderation models examining interactive effects between treatment condition and polysomnographic sleep parameters on symbol-coding performance change from post-stimulation to post-sleep.

| **Outcome: Symbol Code Change** | | |  |  |  |  |  |  |
| --- | --- | --- | --- | --- | --- | --- | --- | --- |
| Intercept | Est. | 0.243 | 0.025 | 0.234 | 0.149 | 0.2816 | -0.384 | 0.2001 |
|  | *CI* | (0.026,0.460) | (-0.284,0.335) | (-0.031,0.499) | (-0.150,0.448) | (0.055,0.508) | (-1.057,0.288) | (-0.038,0.438) |
|  | *df* | 18.337 | 24.327 | 21.151 | 26.711 | 19.309 | 32.607 | 19.3709 |
|  | *t* | 2.350 | 0.169 | 1.833 | 1.024 | 2.602 | -1.163 | 1.7546 |
|  | *p* | 0.030 | 0.867 | 0.081 | 0.315 | 0.017 | 0.253 | 0.0951 |
|  |  |  |  |  |  |  |  |  |
| **Age** | Est. | -0.004 | -0.005 | -0.008 | -0.008 | -0.0062 | -0.004 | -0.007 |
|  | *CI* | (-0.013,0.004) | (-0.013,0.003) | (-0.016, 0.001) | (-0.016, 0.001) | (-0.014,0.002) | (-0.0013,0.005) | (-0.016,0.002) |
|  | *df* | 17.901 | 17.539 | 17.192 | 17.192 | 17.0459 | 17.964 | 19.957 |
|  | *t* | -1.148 | -1.239 | -1.828 | -1.828 | -1.631 | -0.930 | -1.587 |
|  | *p* | 0.266 | 0.232 | 0.085 | 0.085 | 0.121 | 0.365 | 0.128 |
|  |  |  |  |  |  |  |  |  |
| **Active cTBS (1)** | Est. | -0.031 | 0.135 | 0.139 | 0.121 | -0.024 | 0.1914 | 0.101 |
|  | *CI* | (-0.157,0.095) | (-0.078,0.347) | (-0.048,0.326) | (-0.110,0.351) | (-0.137,0.089) | (-0.492,0.876) | (-0.030,0.233) |
|  | *df* | 19.214 | 19.951 | 22.330 | 21.552 | 18.845 | 19.783 | 22.406 |
|  | *t* | -0.514 | 1.324 | 1.540 | 1.089 | -0.447 | 0.584 | 1.595 |
|  | *p* | 0.613 | 0.201 | 0.138 | 0.288 | 0.660 | 0.566 | 0.125 |
|  |  |  |  |  |  |  |  |  |
| **SWS Latency** | Est. | -0.001 |  |  |  |  |  |  |
|  | *CI* | (-0.002,-4e-04) |  |  |  |  |  |  |
|  | *df* | 34.969 |  |  |  |  |  |  |
|  | *t* | -2.740 |  |  |  |  |  |  |
|  | *p* | 0.010 |  |  |  |  |  |  |
|  |  |  |  |  |  |  |  |  |
| **Active cTBS × SWS Latency** | Est. | 0.0012 |  |  |  |  |  |  |
|  | *CI* | (-4e-04,0.0029) |  |  |  |  |  |  |
|  | *df* | 22.497 |  |  |  |  |  |  |
|  | *t* | 1.536 |  |  |  |  |  |  |
|  | *p* | 0.138 |  |  |  |  |  |  |
|  |  |  |  |  |  |  |  |  |
| **SWS Duration** | Est. |  | 0.0019 |  |  |  |  |  |
|  | *CI* |  | (-2e-04,0.0039) |  |  |  |  |  |
|  | *df* |  | 34.764 |  |  |  |  |  |
|  | *t* |  | 1.797 |  |  |  |  |  |
|  | *p* |  | 0.081 |  |  |  |  |  |
|  |  |  |  |  |  |  |  |  |
| **Active cTBS × SWS Duration** | Est. |  | -0.001 |  |  |  |  |  |
|  | *CI* |  | (-0.004,0.002) |  |  |  |  |  |
|  | *df* |  | 20.533 |  |  |  |  |  |
|  | *t* |  | -0.697 |  |  |  |  |  |
|  | *p* |  | 0.494 |  |  |  |  |  |
|  |  |  |  |  |  |  |  |  |
| **REM Latency** | Est. |  |  | 0.000 |  |  |  |  |
|  | *CI* |  |  | (-0.001,0.001) |  |  |  |  |
|  | *df* |  |  | 33.494 |  |  |  |  |
|  | *t* |  |  | -0.042 |  |  |  |  |
|  | *p* |  |  | 0.967 |  |  |  |  |
|  |  |  |  |  |  |  |  |  |
| **Active cTBS × REM Latency** | Est. |  |  | -0.001 |  |  |  |  |
|  | *CI* |  |  | (-0.0025,9e-04) |  |  |  |  |
|  | *df* |  |  | 23.605 |  |  |  |  |
|  | *t* |  |  | -0.960 |  |  |  |  |
|  | *p* |  |  | 0.347 |  |  |  |  |
|  |  |  |  |  |  |  |  |  |
| **REM Duration** | Est. |  |  |  | 0.001 |  |  |  |
|  | *CI* |  |  |  | (-0.002,0.004) |  |  |  |
|  | *df* |  |  |  | 34.946 |  |  |  |
|  | *t* |  |  |  | 0.854 |  |  |  |
|  | *p* |  |  |  | 0.399 |  |  |  |
|  |  |  |  |  |  |  |  |  |
| **Active cTBS × REM Duration** | Est. |  |  |  | -0.001 |  |  |  |
|  | *CI* |  |  |  | (-0.004,0.002) |  |  |  |
|  | *df* |  |  |  | 22.435 |  |  |  |
|  | *t* |  |  |  | -0.591 |  |  |  |
|  | *p* |  |  |  | 0.561 |  |  |  |
|  |  |  |  |  |  |  |  |  |
| **Latency to Persistent Sleep** | Est. |  |  |  |  | -0.0021 |  |  |
|  | *CI* |  |  |  |  | (-0.004,-4e-04) |  |  |
|  | *df* |  |  |  |  | 34.032 |  |  |
|  | *t* |  |  |  |  | -2.464 |  |  |
|  | *p* |  |  |  |  | 0.019 |  |  |
|  |  |  |  |  |  |  |  |  |
| **Active cTBS x Latency to Persistent Sleep** | Est. |  |  |  |  | 0.0021 |  |  |
|  | *CI* |  |  |  |  | (0,0.0041) |  |  |
|  | *df* |  |  |  |  | 19.497 |  |  |
|  | *t* |  |  |  |  | 2.106 |  |  |
|  | *p* |  |  |  |  | 0.048 |  |  |
|  |  |  |  |  |  |  |  |  |
| **Sleep Efficiency** | Est. |  |  |  |  |  | 0.0058 |  |
|  | *CI* |  |  |  |  |  | (-0.001,0.013) |  |
|  | *df* |  |  |  |  |  | 34.399 |  |
|  | *t* |  |  |  |  |  | 1.743 |  |
|  | *p* |  |  |  |  |  | 0.090 |  |
|  |  |  |  |  |  |  |  |  |
| **Active cTBS x Sleep Efficiency** | Est. |  |  |  |  |  | -0.003 |  |
|  | *CI* |  |  |  |  |  | (-0.011,0.006) |  |
|  | *df* |  |  |  |  |  | 19.449 |  |
|  | *t* |  |  |  |  |  | -0.711 |  |
|  | *p* |  |  |  |  |  | 0.486 |  |
|  |  |  |  |  |  |  |  |  |
| **Wake After Persistent Sleep Onset** | Est. |  |  |  |  |  |  | 0.0003 |
|  | *CI* |  |  |  |  |  |  | (-0.002,0.002) |
|  | *df* |  |  |  |  |  |  | 33.364 |
|  | *t* |  |  |  |  |  |  | 0.290 |
|  | *p* |  |  |  |  |  |  | 0.774 |
|  |  |  |  |  |  |  |  |  |
| **Active cTBS × Wake After Sleep Onset** | Est. |  |  |  |  |  |  | -0.001 |
|  | *CI* |  |  |  |  |  |  | (-0.004,0.002) |
|  | *df* |  |  |  |  |  |  | 26.880 |
|  | *t* |  |  |  |  |  |  | -0.855 |
|  | *p* |  |  |  |  |  |  | 0.400 |

Supplementary Table 3.

Moderation models examining interactive effects between treatment condition and polysomnographic sleep parameters on story-recall performance change from post-stimulation to post-sleep.

| **Outcome: Story Change** | | |  |  |  |  |  |  |
| --- | --- | --- | --- | --- | --- | --- | --- | --- |
| Intercept | Est. | -0.005 | -0.005 | 0.036 | 0.040 | 0.0611 | -0.042 | 0.0394 |
|  | *CI* | (-0.142,0.131) | (-0.142,0.131) | (-0.084,0.155) | (-0.114,0.194) | (-0.024,0.147) | (-0.503,0.420) | (-0.043,0.122) |
|  | *df* | 35.378 | 35.378 | 35.500 | 35.842 | 33.629 | 35.671 | 35.996 |
|  | *t* | -0.081 | -0.081 | 0.606 | 0.532 | 1.453 | -0.184 | 0.972 |
|  | *p* | 0.936 | 0.936 | 0.549 | 0.598 | 0.156 | 0.855 | 0.337 |
|  |  |  |  |  |  |  |  |  |
| **Active cTBS (1)** | Est. | 0.069 | 0.069 | 0.549 | 0.134 | -0.014 | 0.030 | 0.057 |
|  | *CI* | (-0.094,0.233) | (-0.094,0.233) | (-0.174,0.130) | (-0.032,0.301) | (-0.109,0.080) | (-0.527,0.588) | (-0.042,0.157) |
|  | *df* | 19.098 | 19.098 | 21.988 | 20.400 | 18.710 | 19.330 | 21.128 |
|  | *t* | 0.885 | 0.885 | -0.301 | 1.683 | -0.320 | 0.114 | 1.193 |
|  | *p* | 0.387 | 0.387 | 0.766 | 0.108 | 0.753 | 0.911 | 0.246 |
|  |  |  |  |  |  |  |  |  |
| **SWS Latency** | Est. | 0.001 |  |  |  |  |  |  |
|  | *CI* | (-0.001,0.0024) |  |  |  |  |  |  |
|  | *df* | 35.759 |  |  |  |  |  |  |
|  | *t* | 0.815 |  |  |  |  |  |  |
|  | *p* | 0.420 |  |  |  |  |  |  |
|  |  |  |  |  |  |  |  |  |
| **Active cTBS × SWS Latency** | Est. | -0.001 |  |  |  |  |  |  |
|  | *CI* | (-0.0029,0.0015) |  |  |  |  |  |  |
|  | *df* | 19.523 |  |  |  |  |  |  |
|  | *t* | -0.698 |  |  |  |  |  |  |
|  | *p* | 0.493 |  |  |  |  |  |  |
|  |  |  |  |  |  |  |  |  |
| **SWS Duration** | Est. |  | 0.001 |  |  |  |  |  |
|  | *CI* |  | (-0.001,0.002) |  |  |  |  |  |
|  | *df* |  | 35.759 |  |  |  |  |  |
|  | *t* |  | 0.815 |  |  |  |  |  |
|  | *p* |  | 0.420 |  |  |  |  |  |
|  |  |  |  |  |  |  |  |  |
| **Active cTBS × SWS Duration** | Est. |  | -0.001 |  |  |  |  |  |
|  | *CI* |  | (-0.003,0.002) |  |  |  |  |  |
|  | *df* |  | 19.523 |  |  |  |  |  |
|  | *t* |  | -0.698 |  |  |  |  |  |
|  | *p* |  | 0.493 |  |  |  |  |  |
|  |  |  |  |  |  |  |  |  |
| **REM Latency** | Est. |  |  | 0.0001 |  |  |  |  |
|  | *CI* |  |  | (-0.001,0.001) |  |  |  |  |
|  | *df* |  |  | 33.537 |  |  |  |  |
|  | *t* |  |  | 0.174 |  |  |  |  |
|  | *p* |  |  | 0.863 |  |  |  |  |
|  |  |  |  |  |  |  |  |  |
| **Active cTBS × REM Latency** | Est. |  |  | 0.0004 |  |  |  |  |
|  | *CI* |  |  | (-0.001,0.0018) |  |  |  |  |
|  | *df* |  |  | 23.177 |  |  |  |  |
|  | *t* |  |  | 0.595 |  |  |  |  |
|  | *p* |  |  | 0.558 |  |  |  |  |
|  |  |  |  |  |  |  |  |  |
| **REM Duration** | Est. |  |  |  | 0.0001 |  |  |  |
|  | *CI* |  |  |  | (-0.002,0.002) |  |  |  |
|  | *df* |  |  |  | 35.268 |  |  |  |
|  | *t* |  |  |  | 0.059 |  |  |  |
|  | *p* |  |  |  | 0.954 |  |  |  |
|  |  |  |  |  |  |  |  |  |
| **Active cTBS × REM Duration** | Est. |  |  |  | -0.002 |  |  |  |
|  | *CI* |  |  |  | (-0.0038,6e-04) |  |  |  |
|  | *df* |  |  |  | 21.108 |  |  |  |
|  | *t* |  |  |  | -1.525 |  |  |  |
|  | *p* |  |  |  | 0.142 |  |  |  |
|  |  |  |  |  |  |  |  |  |
| **Latency to Persistent Sleep** | Est. |  |  |  |  | -0.0004 |  |  |
|  | *CI* |  |  |  |  | (-0.002,0.0012) |  |  |
|  | *df* |  |  |  |  | 34.879 |  |  |
|  | *t* |  |  |  |  | -0.521 |  |  |
|  | *p* |  |  |  |  | 0.605 |  |  |
|  |  |  |  |  |  |  |  |  |
| **Active cTBS x Latency to Persistent Sleep** | Est. |  |  |  |  | 0.001 |  |  |
|  | *CI* |  |  |  |  | (-9e-04,0.0025) |  |  |
|  | *df* |  |  |  |  | 19.2105 |  |  |
|  | *t* |  |  |  |  | 0.949 |  |  |
|  | *p* |  |  |  |  | 0.355 |  |  |
|  |  |  |  |  |  |  |  |  |
| **Sleep Efficiency** | Est. |  |  |  |  |  | 0.001 |  |
|  | *CI* |  |  |  |  |  | (-0.004,0.007) |  |
|  | *df* |  |  |  |  |  | 35.372 |  |
|  | *t* |  |  |  |  |  | 0.452 |  |
|  | *p* |  |  |  |  |  | 0.654 |  |
|  |  |  |  |  |  |  |  |  |
| **Active cTBS x Sleep Efficiency** | Est. |  |  |  |  |  | 0.0003 |  |
|  | *CI* |  |  |  |  |  | (-0.006,0.007) |  |
|  | *df* |  |  |  |  |  | 19.103 |  |
|  | *t* |  |  |  |  |  | 0.079 |  |
|  | *p* |  |  |  |  |  | 0.938 |  |
|  |  |  |  |  |  |  |  |  |
| **Wake After Persistent Sleep Onset** | Est. |  |  |  |  |  |  | 0.0001 |
|  | *CI* |  |  |  |  |  |  | (-0.001,0.002) |
|  | *df* |  |  |  |  |  |  | 33.066 |
|  | *t* |  |  |  |  |  |  | 0.169 |
|  | *p* |  |  |  |  |  |  | 0.867 |
| **Active cTBS × Wake After Sleep Onset** | Est. |  |  |  |  |  |  | -0.001 |
|  | *CI* |  |  |  |  |  |  | (-0.003,9e-04) |
|  | *df* |  |  |  |  |  |  | 24.221 |
|  | *t* |  |  |  |  |  |  | -1.155 |
|  | *p* |  |  |  |  |  |  | 0.260 |

Supplementary Table 4.

Longitudinal main effects of treatment condition across three timepoints each visit, by treatment condition, and interactions between timepoint and treatment condition.

|  |  | **Digit Span** | **Symbol Coding** | **Story Recall** |
| --- | --- | --- | --- | --- |
| (Intercept) | Estimate (B) | 11.459 | 76.599 | 11.811 |
|  | *CI* | (7.3614,15.5565) | (54.7647,98.4333) | (8.7956,14.8273) |
|  | *df* | 17.837 | 17.629 | 17.952 |
|  | *t* | 5.879 | 7.382 | 8.230 |
|  | *p* | < .0001 | < .0001 | < .0001 |
|  |  |  |  |  |
| **Age** | Estimate | -0.020 | -0.887 | -0.050 |
|  | *CI* | (-0.1849,0.1448) | (-1.7676,-0.007) | (-0.1714,0.071) |
|  | *df* | 17 | 7 | 17 |
|  | *t* | -0.256 | -2.127 | -0.874 |
|  | *p* | 0.801 | 0.048 | 0.395 |
|  |  |  |  |  |
| **Sex (Male =1)** | Estimate | 1.5744 | 6.9252 | -0.028 |
|  | *CI* | (-0.5775,3.7262) | (-4.5651,18.4155) | (-1.6103,1.5536) |
|  | *df* | 17 | 17 | 17 |
|  | *t* | 1.5436 | 1.2716 | -0.038 |
|  | *p* | 0.1411 | 0.2206 | 0.970 |
|  |  |  |  |  |
| **Post-cTBS** | Estimate | -0.200 | 6.050 | -1.750 |
|  | *CI* | (-1.1245,0.7245) | (1.7654,10.3346) | (-2.4746,-1.0254) |
|  | *df* | 95 | 95 | 95 |
|  | *t* | -0.4295 | 2.8033 | -4.795 |
|  | *p* | 0.6686 | 0.0061 | < .0001 |
|  |  |  |  |  |
| **Post-Sleep** | Estimate | 0.100 | 8.100 | -1.300 |
|  | *CI* | (-0.8245,1.0245) | (3.8154,12.3846) | (-2.0246,-0.5754) |
|  | *df* | 95 | 95 | 95 |
|  | *t* | 0.2147 | 3.7531 | -3.562 |
|  | *p* | 0.8304 | 0.0003 | 0.0006 |
|  |  |  |  |  |
| **Active cTBS (1)** | Estimate | 0.050 | 1.400 | -0.150 |
|  | *CI* | (-0.8745,0.9745) | (-2.8846,5.6846) | (-0.8746,0.5746) |
|  | *df* | 95 | 95 | 95 |
|  | *t* | 0.1074 | 0.649 | -0.411 |
|  | *p* | 0.9147 | 0.518 | 0.682 |
|  |  |  |  |  |
| **Post-cTBS x Active cTBS (1)** | Estimate | -0.200 | -0.100 | 0.150 |
|  | *CI* | (-1.5075,1.1075) | (-6.1593,5.9593) | (-0.8747,1.1747) |
|  | *df* | 95 | 95 | 95 |
|  | *t* | -0.3037 | -0.033 | 0.291 |
|  | *p* | 0.762 | 0.974 | 0.772 |
|  |  |  |  |  |
| **Post-Sleep x Active cTBS(1)** | Estimate | -0.150 | 2.950 | 0.35 |
|  | *CI* | (-1.4575,1.1575) | (-3.1093,9.0093) | (-0.6747,1.3747) |
|  | *df* | 95 | 95 | 95 |
|  | *t* | -0.2278 | 0.967 | 0.678 |
|  | *p* | 0.820 | 0.336 | 0.499 |

Supplementary Table 5.

Individual-level total scores on the Insomnia Severity Index (ISI), Pittsburg Sleep Quality Index (PSQI), and the Epworth Sleepiness Scale (ESS) as well as inclusion cutoffs (0 or 1) used at enrollment.

|  | **Insomnia Severity** | | **Sleep Disturbances** | | **Daytime Sleepiness** | |
| --- | --- | --- | --- | --- | --- | --- |
| ID #: | ISI ≥ 15 | ISI Total | PSQI ≥ 6 | PSQI Total | ESS ≥ 11 | ESS Total |
| 1 | 1 | 16 | 1 | 9 | 1 | 14 |
| 2 | 1 | 21 | 1 | 12 | 0 | 9 |
| 3 | 0 | 7 | 1 | 7 | 1 | 12 |
| 4 | 1 | 18 | 1 | 9 | 1 | 14 |
| 5 | 1 | 17 | 1 | 8 | 0 | 10 |
| 6 | 1 | 15 | 1 | 10 | 0 | 8 |
| 7 | 1 | 18 | 1 | 13 | 1 | 12 |
| 8 | 1 | 19 | 1 | 13 | 1 | 13 |
| 9 | 1 | 15 | 1 | 14 | 1 | 13 |
| 10 | 1 | 17 | 1 | 13 | 0 | 7 |
| 11 | 1 | 20 | 1 | 13 | 1 | 15 |
| 12 | 1 | 26 | 1 | 14 | 1 | 15 |
| 13 | 0 | 14 | 1 | 8 | 1 | 13 |
| 14 | 1 | 17 | 1 | 14 | 0 | 10 |
| 15 | 1 | 16 | 1 | 12 | 0 | 3 |
| 16 | 1 | 16 | 1 | 12 | 1 | 12 |
| 17 | 1 | 24 | 1 | 16 | 1 | 12 |
| 18 | 1 | 17 | 1 | 11 | 1 | 14 |
| 19 | 1 | 18 | 1 | 14 | 0 | 3 |
| 20 | 1 | 15 | 1 | 9 | 0 | 3 |
|  |  |  |  |  |  |  |
